# Should Portasystemic Shunts be Avoided in Potential Liver Transplant Candidates?

**DOI:** 10.1155/1990/58079

**Published:** 1990

**Authors:** Jean C. Emond, C. Broelsch

**Affiliations:** The University of Chicago Department of Surgery 5841 South Maryland Avenue Chicago Illinois 60637 USA


### SHOULD PORTASYSTEMIC SHUNTS BE AVOIDED IN POTENTIAL LIVER TRANSPLANT CANDIDATES?

### ABSTRACT

*Brems, J.J., Hiatt, J.R., Klein, A.S., Millis, J.M., Colonna, J.O., Quinones-Baldrich, W.J., Ramming, K.P. and Busuttil, R.W. (1989) Effect of a prior portasystemic shunt on subsequent liver transplantation. Annals of Surgery, 209, 51–56*

**Fifteen patients who had a prior portasystemic shunt underwent orthotopic liver transplantation. Shunt types were portacaval in six patients, II-graft mesocaval in six, distal splenorenal in two, and proximal splenorenal in one. Mean blood loss and hospital stay were highest in the portacaval group. Retransplants (two patients) and deaths (two patients) also were limited to this group. In this report, technical considerations, advantages, and disadvantages of the various shunt types are described. Management of patients with late stages of portal hypertension must include estimation of the effects of a portasystemic shunt on subsequent liver transplantation. It is concluded that portacaval shunts should be avoided in patients who may be considered for transplantation. Distal splenorenal shunts are best performed in younger patients with intractable variceal bleeding who are not expected to require transplantation in the near future. A mesocaval II-graft is the shunt of choice in patients who are current liver transplant candidates.**

### PAPER DISCUSSION

KEY WORDS: Portacaval shunt, liver transplantation, portal hypertension

The recent publication from Brems, J., *et al.*[1] underscores the importance of clinical investigation into the relationship between the treatment of variceal bleeding and the practice of hepatic transplantation. This deficit is due to two problems in clinical hepatology. First, there has been a lack of involvement by many transplant groups in the pre-transplant management of patients with liver disease. Second, groups investigating the treatment of portal hypertension, even in recent prospective trials[2], have not considered the importance of OLT either as an endpoint in the failure of other treatment for portal hypertension, or as one of the available treatments for portal hypertension.

In the report from UCLA the authors evaluate the results of OLT in a subgroup of 15 patients with prior porto-systemic shunting. The authors are to be congratulated for their excellent results in performing OLT in this technically challenging subgroup of candidates. Until recently these patients were excluded as candidates by some centers regarding such surgery as technical contra-indications.

The authors experienced extreme technical difficulties, and poor results in the subgroup with previous porto-caval shunt, and therefore, recommended that this operation be avoided in potential transplant candidates. While this is a reasonable recommendation, it is not clear that their data permit this conclusion. The authors do not clarify whether the shunt operations were performed by their own surgical team as part of the management of the pre-transplant complications. They also fail to discuss the role of the underlying liver disease (chronic hepatitis was the predominant diagnosis in the porto-caval shunt group). It has recently become clear that liver transplantation for parenchymal cirrhosis is associated with greater technical difficulties, blood loss, and a higher mortality than cholestatic cirrhosis. In addition, the clinical context prior to shunting usually influences the choice of the procedure. Ascites, regarded as a contra-indication to selective shunting, has been identified as a factor associated with poor prognosis in both the natural history of liver disease and with increase risk after OLT. It is therefore likely that the patient receiving porto-caval shunts had more severe liver disease and a higher risk for OLT.

Based on a recent report evaluating the incidence of previous variceal bleeding in 1000 liver transplants at the University of Pittsburgh, the authors recommended abandonment of portosystemic shunting as an option in the treatment of liver disease[3]. The use of retrospective data to make these recommendations was seriously questioned by Wexler in the discussion published with the report.

Clarification of the role of hepatic transplantation and portal decompression will require the prospective analysis of populations with portal hypertension, not the retrospective evaluation of a subgroup of liver transplant recipients previously treated for variceal bleeding. Equally important, prospective study of portal hypertension therapy must include liver transplantation both as an option in the management of variceal bleeding, and as an endpoint in patients with treatment failure after sclerosis or shunt therapy.

At the present time, we feel it is more appropriate to consider shunt surgery as one of the palliative options in most patients with liver disease. Shunts should therefore, be performed with the consideration of future transplant therapy in which avoidance of the hepatic hilus is probably advisable. It should not be forgotten, however, that shunt surgery is potentially curative therapy in non-progressive or potentially reversible liver disease and will continue to have a role in modern surgery[4].

Jean C. Emond, MD
C. Broelsch, MD
The University of Chicago
Department of Surgery
5841 South Maryland Avenue
Chicago, ILLINOIS 60637
U.S.A.

### REFERENCES

1. Brems, J.J., Hiatt, J.R., Klein, A.S., Millis, J.M., Colonna, J.O., Quinones-Baldrich, W.J., Ramming, K.P. and Busuttil, R.W. (1989) Effect of a prior portasystemic shunt on subsequent liver transplantation. *Annals of Surgery*, **209**, 51–56
2. Terblanche, J., Burroughs, A.K. and Hobbs, K.E.F. (1989) Medical progress: Controversies in the management of bleeding varices. *New England Journal of Medicine*, **320**, 1393–1398, 1469–1474
3. Iwatsuki, S., Starzl, T.E., Todo, S. *et al.* (1988) Liver transplantation in the treatment of bleeding esophageal varices. *Surgery*, **104**, 697–705
4. Broelsch, C.E., Vogelbach, P., Emond, J.C., Thislethwaite, J.R., Woodle, S.A., Baker, A.L. and Whitington, P.F. (Kongr. 1989) Shuntchirurgie im hinblick auf die indikation zur lebertransplantation. Langenbecks Arch Chir Suppl II, 293–299

Lund, May 13th-17th, 1991

**9th International Course on Advanced Hepato-Pancreato-Biliary Surgery**

Philip Sandblom lecturer:

Professor Arthur Li
Hongkong

- Pathophysiology
- Work-up
- Operations
- Follow-up
- Non-operative techniques
- Radiology
- Complications-Lectures
- Videos and Films
- Discussions

For further information, please contact:
Professor Stig Bengmark
PO Box 5003
S-220 05 LUND 5, Sweden

